# The constrained disorder principle accounts for quantum effects in biological systems

**DOI:** 10.25122/jml-2025-0109

**Published:** 2026-01

**Authors:** Yaron Ilan

**Affiliations:** 1 Department of Medicine, Hadassah Medical Center, Faculty of Medicine, Hebrew University, Jerusalem, Israel

**Keywords:** Quantum biology, digital health, complex systems, variability, randomness, constrained disorder CDP, Constrained Disorder Principle

## Abstract

The constrained disorder principle (CDP) defines all systems in the universe by their inherent variability and underlies their proper functioning. According to this principle, variability underlies every biological process and is fundamental to the proper functioning of systems. The CDP explains that internal and external noise is necessary for biological systems to function appropriately, provided it is kept within dynamic boundaries, enabling adaptation to perturbations. Many phenomena in biological systems are complex to describe using current rules, but can be explained by quantum effects. The paper discusses data on quantum randomness, which may be attributed to the CDP. It describes CDP-based second-generation artificial intelligence systems that introduce variability into biological systems to enhance their functionality. The paper outlines the potential of utilizing CDP-based quantum randomness for various applications, including leveraging quantifiable variables of quantum randomness to address malfunctions in biological processes and enhance the efficiency of biological systems.

## Introduction

Many phenomena in biological systems are complex to explain using current rules. Therefore, it is suggested that quantum effects may underlie some of their functions [1]. Schrödinger was among the first to propose a connection between quantum and biological processes [2-4]. He examined the laws of classical physics and chemistry, particularly those of thermodynamics, which he referred to as “order from disorder” laws. He suggested that their apparent orderliness arises from the underlying disorder of molecular movements. Schrödinger sought to explain how living organisms challenge the second law of thermodynamics, which states that the universe tends to become more disordered over time. He argued that living systems can bypass decoherence, aligning with the idea that environmental noise helps maintain coherence within living cells [3,5].

Biological systems are more complex than standard physical systems, making it difficult to distinguish 'pure' quantum effects from classical processes also at play. Additionally, biological organisms function as non-linear open systems that operate far from thermodynamic equilibrium while remaining close to steady states [6].

An interpretation of quantum mechanics seeks to explain how the mathematical theory relates to our experienced reality. There is an ongoing debate about whether all objects are subject to randomness. Several schools of thought exist, each with varying perspectives on whether quantum mechanics is deterministic or stochastic, and whether it is local or non-local. They also differ on which aspects of quantum mechanics are natural, the nature of measurement, and related topics [7,8].

The question of whether quantum effects influence biological systems need not be addressed directly. However, it remains unclear how quantifiable these effects are in terms of their influence. There is a need to describe the underlying mechanisms of biological function mathematically, incorporate realistic quantum fluctuations, and analyze their quantitative impact. This approach may suggest that, when considered across large populations, these effects are of a magnitude sufficient to be observable at the macroscopic scale.

The constrained disorder principle (CDP) is a framework that defines systems and underlies the proper functioning of biological systems in terms of their degree of inherent variability. It attempts to provide a platform for explaining how both internal and external noises are necessary for the proper functioning of these systems [9]. This paper critically examines the potential quantum effects in biological systems, highlighting the significant role that CDP may play in these phenomena. It further explores the potential to leverage CDP-based quantum variability to enhance the functionality of biological processes.

### The constrained disorder principle defines systems and explains the variability of biological processes

The CDP defines systems by their inherent variability, which is crucial for the proper functioning of complex systems. This variability is essential for systems to operate effectively within dynamic boundaries. It accounts for the constant changes in internal and external conditions that biological processes face, enabling continuous adaptation [8,9].

Variability is a fundamental characteristic of biological systems, spanning from the genomic level to cellular functions and entire organ systems. The concept of the CDP posits that these variations are not merely commonplace in biological processes but, in fact, vital to their optimal functioning. Embracing this variability can significantly enhance system performance [10,11]. The CDP emphasizes that the effectiveness of a biological system depends on its ability to harness a dynamic range of variability, ultimately driving improved functionality and resilience. When a system malfunctions, it is usually due to inadequate regulation of variability under certain conditions, which can either be excessive or insufficient variability [12-15].

### Making use of noise in biological systems based on the CDP

The CDP highlights the critical role of variability in ensuring the optimal functioning of biological systems. This inherent biological variability is fundamental to the intricate relationships between environments and systems. As a result of the CDP, we have witnessed the emergence of second-generation artificial intelligence (AI) systems designed to rectify malfunctions in biological processes. These advanced systems achieve this by strategically incorporating constrained noise, fundamentally enhancing the reliability and efficiency of biological interactions [16].

An example is the use of second-generation AI systems to overcome drug resistance and enhance drug efficacy [9,12,13,15-23]. Patients with chronic diseases often suffer from a loss of response to their medications. Multiple reasons underlie the partial or complete loss of drugs' effectiveness, affecting 30-50% of patients with most chronic diseases [24]. Second-generation AI systems are designed to implement variability in dosing and drug administration times, aiming to overcome drug resistance.

The CDP-based AI system utilizes biological noise and is being developed in three steps. In the first step, variability in dosing and drug administration timing is introduced into treatment regimens, while keeping them within predefined ranges. In the second step, a closed-loop algorithm determines the degree of variability in the outcome, enabling personalized output. In the third step, quantified variability signatures—such as heart rate variability, glucose variability, and others—are incorporated into the algorithm to improve its accuracy and biological relevance [9,12,13,16-19].

This method is applied to patients with multiple diseases and has been shown to improve clinical outcomes [9,12,13,16-22,24-53]. It illustrates how regulated noise can enhance functionality.

Using the CDP-based AI system, introducing regulated variability into treatment regimens was shown to be effective for patients with chronic heart failure, multiple sclerosis, cancer, genetic diseases, and chronic pain [13,20-23]. In these studies, integrating randomness into treatment plans via a CDP-based AI system was associated with improved clinical and laboratory outcomes. This concept can be applied across different disease states, demonstrating that controlled variability can enhance functionality and address system malfunctions [21,25-52,54-59].

### Quantum effects are detected in biological systems

Quantum theory is based on the concept that matter exhibits both wave-like and particle-like properties, and that waves can exhibit particle-like properties. There is ongoing debate about whether measurements have an “ontological” significance—relating to the nature of existence—or an “epistemological” significance—relating to the nature of knowledge. It raises questions about whether measurements describe an external reality or reflect our understanding of the world. Despite this debate, measurement remains valuable and valid for many practical applications [60,61].

Quantum theory explains how delocalized objects evolve, but when a measurement is made, it introduces randomness and marks a break in this evolution. This interruption of the quantum state's wave-like propagation is referred to as the “collapse of the wave function.” When a 50/50 beam splitter divides a photon beam, it randomly directs the beam to one of two detectors, resulting in a completely random sequence of zeros and ones [62,63].

The CDP framework may align with the fundamental concepts of quantum physics, including wave-particle duality, superposition, entanglement, coherence, nonlocality, and tunneling [64,65].

Superposition is a property of quantum particles that allows them to exist in multiple locations simultaneously within a probability zone. It means that a particle can occupy all possible positions simultaneously. The principle of superposition is related to both determinism and quantum randomness. In a wave equation, the shape and position of an interference pattern are precisely determined, while the square of the wave's amplitude indicates the likelihood of detecting a particular event. However, each event is inherently random, occurring within defined probabilities [64,65].

Entanglement refers to a property of two particles, allowing them to instantaneously know the state of each other when one is observed, regardless of the distance between them. A quantum state can preserve its entanglement and superposition for a limited time despite interactions and thermalization [66,67]. Tunneling is a phenomenon in which a particle can pass through a potential energy barrier and appear on the other side, even if it lacks the necessary kinetic energy to overcome that barrier [68].

In certain conditions, many particles cannot be described by separate wave functions for each one. Instead, the system is represented by a single wave function that describes its collective behavior. This phenomenon, known as quantum coherence, occurs when individual particles lose their distinct identities, causing the entire system to behave as a unified entity [6]. Coherence enhances transportation in complex, disordered systems [69]. Entanglement arises from superposition, which is fundamental to coherence [70].

The CDP emphasizes that complex systems must balance chaos and order to function effectively. When this balance is achieved, it can significantly enhance their coherence time [68]. When measurements are made on a single particle, the entire wave function of the system collapses, resulting in an immediate effect on all particles, regardless of their spatial location. This inherently quantum-mechanical interaction over distance is known as non-local quantum entanglement [6].

Since all living organisms are composed of atoms that follow the principles of quantum physics, it follows that quantum physics may also influence biological processes. Quantum effects are crucial for understanding certain biological phenomena that classical physics cannot account for. Living systems utilize quantum mechanics to accomplish tasks that are not possible under classical rules [71]. In alliance with the CDP, it was proposed as a mechanism for enhancing the efficiency of these processes, outperforming the best classical equivalent [66].

In laboratory settings, quantum states cannot be directly observed; only observables can be measured. It makes it challenging to account for specific biological processes governed by quantum rules [72,73]. Quantum properties influence essential biological processes, including DNA stability, neuronal function, mitochondrial enzyme catalysis, avian navigation, olfaction, light-harvesting in photosynthesis, and magnetic-field navigation in birds [74]. Quantum effects are believed to be diminished by thermal decoherence at scales larger than those of atoms or subatomic particles. It occurs at higher temperatures and in aqueous environments, creating a 'noisy' backdrop that affects molecular interactions [75].

These examples align with the CDP, showing that variability is crucial for these processes. However, additional data is necessary to support this association.

### The CDP may account for quantum randomness

Thermodynamics and quantum physics can be viewed as theories of randomness [72]. The CDP may account for quantum randomness in biological systems [9,76].

The thermodynamic concept is grounded in the second law of thermodynamics, which states that the entropy of an isolated system can only increase over time. This increase in entropy corresponds to the information lost when transitioning from the microscopic to the macroscopic scale [72,77]. Classical thermodynamics views randomness and irreversibility as limitations resulting from a lack of knowledge about physical states. The ultimate description of physical reality is found at the microscopic level, where randomness disappears, and physical laws are reversible [77,78]. Achieving the highest level of physical reality means that randomness disappears and the laws of physics become reversible. When random disturbances are absent, the system can be perfectly regulated and returned to its initial state. However, any random disturbance can hinder this reversible transformation. In this context, the second law of thermodynamics can be reinstated, providing adequate methods to measure irreversibility through the amount of entropy produced [72].

According to the CDP, randomness is essential for the system's functionality and occurs at all levels in varying degrees, while being maintained within dynamic boundaries. The degree of randomness is dynamic and changes in response to perturbations of both internal and external environments [9].

Statistical physics involves searching for hidden variables equivalent to micro-states and eliminating the measurement postulate [79]. The repeated violations of Bell's inequalities have ruled out local hidden variable theories. This perspective suggests that the world consists of elementary systems with hidden states and wave functions that evolve according to the deterministic Schrödinger equation [72]. The “missing laws” that could explain all biological phenomena have been proposed as those related to chance and probability in the quantum world. Non-living objects are governed by the average random motion of millions of particles, meaning that the motion of a single molecule does not significantly affect the entire object. In contrast, a few specific molecules that control the dynamics of living cells can exert significant influence, amplifying quantum-level events that affect their motion and ultimately the entire organism [4].

According to the CDP, the inherent variability that characterizes systems in the microscopic and macroscopic worlds may explain many previously unexplained phenomena [10,11].

Quantum randomness is a fundamental source of irreversibility arising from stochastic quantum measurements [72,78,80,81]. Randomness is a unique characteristic of quantum phenomena, and the outcomes of quantum measurements are distributed randomly [82,83]. Historically, quantum randomness has been understood as the fundamental disturbance caused by measuring a quantum state. However, this measurement-induced disturbance may render quantum randomness comparable to classical randomness. In both scenarios, the actual states remain concealed at the microscopic level, and information about these states is lost when they transition to the macroscopic realm. From this perspective, quantum measurement can be viewed as an effort to uncover hidden variables and to resolve the 'measurement problem' [72,84].

Stochastic thermodynamics extends the principles of thermodynamics to small, quantum physical systems driven out of equilibrium by an external operator [77]. A stochastic term in the equation effectively describes this interaction and accounts for the system's evolution. This perspective represents a stochastic trajectory in its phase space, consisting of continuous evolution sequences interrupted by stochastic jumps [72]. The specific reasons for randomness in stochastic thermodynamics may not be particularly significant. Methods of stochastic thermodynamics rely on the concept of stochastic trajectories and are applied to various sources of randomness. Quantum randomness can arise from defining particular statistical boundary conditions and can be dynamic. Additionally, it may result from randomly selecting an initial particle configuration with the initial wave function [85].

The perception of randomness can vary based on our knowledge of events [69]. Quantum randomness may rely more on human agents than on objective physical behavior [81]. It is essential to clarify what it means for the results of quantum measurements to be randomly distributed and how to gather empirical evidence for their randomness. Even an ideal observer operating under perfect conditions may never obtain empirical evidence to support the belief that the outcomes of quantum-mechanical experiments are inherently random [85].

The CDP defines every system as inherently constrained by disorder, independent of measurements [10,11]. It suggests that systems are characterized by randomness and that the technical variability of measurements needs to be distinguished from the inherent variability of microscopic and macroscopic systems [19]. It requires distinguishing between measurement-related variability and the intrinsic variability of systems. This distinction can be challenging, as variability is subject to continuous change [10,11,19,54].

Based on the CDP, quantum randomness is similar to thermodynamic randomness, which characterizes systems. It implies that the rules of physics, including those of quantum mechanics, may be part of the constraints on the disorder that describe systems and are dynamically regulated. Nature is fundamentally random, a point commonly accepted in reconstructions of quantum mechanics and an essential part of generalized probability theories [55-57,84].

### The CDP underlies quantum randomness in biological systems

The CDP describes cellular systems as having inherent randomness that is mandatory for proper function. Creating “internal order” is not the primary goal of these systems; instead, it is a result of the dynamic boundaries applied to enhance their function in changing environments. What seems to be an orderly system may be a sum of multiple disordered processes, each constrained by dynamic boundaries [10,14,86-98].

Numerous examples illustrate how quantum effects relate to the fundamental disorder present in biological systems [76,99-102]. Entropy, a measure of randomness, indicates that “tailored randomness” may enhance health by restoring biological complexity [103]. This concept aligns with the CDP, which suggests that a certain degree of disorder is essential for the cell's proper functioning. Furthermore, when appropriately confined, disorder represents actual order [13,86-89]. It applies to living and non-living systems [55-59].

The principle of energy minimization in living systems influences physical processes [6,14]. The discretization of electron energies influences chemical stability, reactivity, bonding, and cellular structure [2,104]. Instead of avoiding dissipation, nature utilizes it by engineering site energies and excitonic coupling to guide energy transport [75].

Cells are self-organizing dissipative structures that require a boundary, an energy inflow, and an entropy outflow. While cells exist far from thermodynamic equilibrium, they function as thermodynamically semi-open systems, acting as machines that utilize energy to establish internal order [103]. The classical view is that for a cell to function correctly, its components must be correctly located at the correct times. Energy is essential for a cell to maintain this order. If a cell's energy levels decrease, it can disrupt cellular organization and function, ultimately leading to cell death. As cells grow more prominent, maintaining this order becomes increasingly challenging [105].

The CDP provides a platform that may help understand the importance of variability in enhancing energy efficiency. Based on this principle, the apparent constrained disorder in biological systems represents an order that helps conserve energy during dynamic perturbations [9,14].

The Fenna-Matthews-Olson (FMO) complex in photosynthesis facilitates efficient energy transfer between the light-harvesting antenna and the reaction center via quantum-mechanical processes. This energy transport occurs randomly, moving incoherently from one FMO complex to another [106]. Nevertheless, the random motion does not account for the nearly perfect efficiency of light transfer, indicating that the energy exists in a quantum superposition state. It allows the energy to travel along all possible pathways simultaneously [34]. The exciton wave function exists in a coherent superposition state across the molecules. This state helps optimize the time required for absorbed light energy to reach the reaction center. It allows energy to consistently take the most efficient path. This example is consistent with the CDP, which recognizes that random energy fluctuations are crucial for improving efficiency [9].

Enzymes catalyze essential reactions for survival that typically cannot occur under normal body conditions, and many of their pathways appear random [107]. Redox reactions are essential to the body's energy-generating metabolic processes. These reactions occur at the binding sites of oxidative phosphorylation within mitochondria. The energy released by these reactions is used to generate a proton gradient, which in turn powers ATP synthesis. Proton transfer plays a critical role in several enzyme-catalyzed reactions. Protons move from one molecule to another through a phenomenon known as quantum tunneling, in which a particle passes through an energy barrier rather than accumulating enough energy to 'climb' over it [74,108].

Variability is fundamental to the typical structure and function of the genome [109]. Classical mechanics is insufficient to explain the structure and stability of DNA [101] adequately. The two strands of the DNA double helix are linked by hydrogen bonds formed by subatomic particles called protons found in the nuclei of hydrogen atoms. These hydrogen bonds create interactions between the bases of the two strands and contribute to the sugar-phosphate backbone that makes up the double helix [110,111]. The stability of the DNA structure is partly due to the energy-level spacing of the molecules involved in DNA replication being larger than the thermal and other disturbances. It helps to ensure that the replication process remains stable. It is suggested that quantum mechanical effects play a role in holding DNA together at room temperature [112,113]. DNA can be visualized as a harmonic oscillator, with a negatively charged electron cloud moving back and forth relative to the nucleotides. The electron clouds surrounding the nucleotides oscillate in opposite directions to maintain the stability of the DNA molecule. These oscillations can exist in a superposition state and may also become entangled. H-transfer reactions involve some degree of proton tunneling due to the small mass of protons and their inherent positional uncertainty [114-116].

Darwinian evolutionary theory posits that mutations occur randomly and that evolutionary changes arise from the selection of advantageous mutations that provide a survival advantage. The DNA bases adhere to strict rules regarding how they bond with one another. However, a slight change, such as a hydrogen bond, can disrupt the pairing rules. As a result, incorrect bases may become linked, leading to a mutation [110]. The hydrogen ions can temporarily shift positions, jumping from their usual site on one side of an energy barrier to the other, in a process known as tautomerism that involves quantum tunneling [117]. The tautomerization process involves the relocation of protons and follows the laws of quantum mechanics [66,116-118]. Suppose this occurs right before the two strands are separated in the initial step of the copying process. In that case, the error can propagate through the cell's replication machinery, leading to an adaptive mutation [110]. Quantum effects play a crucial role in explaining various fundamental biological processes. Decoherence cannot adequately account for the quantum effects observed in DNA structure and function, and in enzymatic reactions that operate at least partly according to quantum rules [119].

These examples demonstrate how quantum effects can mitigate disorder by regulating energy transfer. According to the CDP, these quantum effects may regulate the degree of disorder in biological systems, thereby increasing efficiency and facilitating appropriate adaptations. This variability is mandatory and serves as a mechanism for adapting to internal and external conditions. It also functions as an energy-conserving mechanism [10,11,13,14].

### The CDP may account for quantum uncertainty

The laws of physics are not necessarily violated in living organisms [57]. Instead, life uses the uncertainty principle to increase the probability of certain events occurring. The uncertainty principle indicates that predicting a quantity's rate with complete certainty is generally impossible, even when all initial conditions are known. Macroscopic biological phenomena can be triggered by the behavior of relatively small numbers of particles, which is influenced by complex quantum uncertainty [2,120]. In quantum mechanics, the uncertainty principle—known as Heisenberg's uncertainty principle—consists of various mathematical inequalities that define a limit on the accuracy with which specific pairs of physical quantities of a particle, such as position and momentum, can be predicted from initial conditions [66,74]. The Heisenberg uncertainty principle limits the fidelity of all molecular processes, indicating that predicting a quantity's value with absolute certainty is impossible, even when all initial conditions are specified [6]. Quantum mechanics imposes limits due to the Heisenberg uncertainty principle, which affects the accuracy of collective molecular interactions [6].

The uncertainty principle restricts the degree to which conjugate properties can be simultaneously well-defined. The mathematical framework of quantum physics does not support the idea that a single value can simultaneously and precisely express conjugate properties [112-114].

The CDP conceptually views uncertainty as an inherent aspect of a system's disorder. It states that uncertainty plays a significant role in the dynamic nature of disorder boundaries. These boundaries, which limit the disorder, contribute to our ability to observe the system's behavior. Both the disorder and its boundaries are essential for the proper functioning of systems. The limits imposed by quantum uncertainty are part of the system's constraints, ensuring correct operation [10,11,13].

### The CDP suggests the quantum effects that impact the connections between different environments and systems

The CDP suggests that both external and internal noise are essential for functioning biological systems [24]. Biological systems constantly interact with their environment; environmental signals are critical for initiating and sustaining processes. A certain level of environmental noise is essential for optimizing interactions within biological systems, aligning with the concept of constrained noise, as described in CDP [62,63,121].

Quantum information theory suggests that environmental noise in stationary, non-equilibrium systems can support quantum coherence. It enables the dynamics of living systems to behave more mechanically, in contrast to the thermodynamic behavior that all systems tend to exhibit as the temperature approaches absolute zero. In this context, the molecular disorder is diminished [2,122]. In environmental noise, the associations that characterize quantum effects can become scrambled, transforming pure quantum states into irregular mixtures and indicating a shift from quantum to classical behavior.

This concept suggests that effective noise filtering is necessary to maintain functional integrity. Decoherence is thought to occur when a system interacts with its environment in an irreversible thermodynamic manner, causing different particles involved in a quantum superposition to cease interfering with one another. Decoherence is a significant factor in criticisms of quantum mechanics, as it is used to describe macroscopic biological systems. It emphasizes that only processes occurring on timescales similar to or shorter than environmental interactions can endure long enough to avoid decoherence [6,123,124]. The CDP views all forms of noise as part of the universe. Systems are crafted to adapt to noisy environments, while dynamic boundaries regulate the noise levels within the system [10,11,14,86-98].

Atoms and molecules are part of the conditions that act upon them; they can be viewed as part of each other's environment. Once a sequence of exogenous events occurs, the environment may be perceived differently by participating atoms and molecules. If there is no environmental noise, nothing can assist in conserving energy from the environment [6,125]. The CDP introduces a concept to improve energy usage by utilizing noise. It suggests that instead of resisting the noisy internal and external environments, which consume energy, systems can harness noise to conserve energy [89].

According to the CDP, external and internal noise may be necessary for proper functioning, provided they remain within certain limits. These limits are dynamic and can change at any time [9,12,13,16-19]. Decoherence, which may affect specific biological processes, does not contradict the CDP; instead, it underscores the need to regulate noise rather than eliminate it. Per the CDP, the interactions between a system and its noisy environment do not “scramble” quantum effects. Instead, these interactions are considered part of the expected characteristics of quantum behavior and act as constraining forces on biological processes [9,13,19].

The phase-averaging argument suggests that quantum interference relies on maintaining phase coherence. However, short wavelengths are easily disrupted in changing environments. Fluctuations in geometry, electromagnetic fields, or chemical environments can quickly alter the conditions for constructive and destructive interference. In many cases, thermal volatility makes it challenging to observe interference effects when examining large groups of molecules. It does not imply that quantum mechanics is irrelevant to biology; stochastic dephasing due to destructive quantum interference has been proposed as a mechanism underlying rapid energy transfer in the photosynthetic complex [65,126,127]. These concepts resonate with the CDP and emphasize the importance of accounting for the inherent noise of systems.

### Exploring the potential of quantum systems to utilize randomness and improve performance with the CDP

Quantum effects can introduce randomness and enhance various functions, as demonstrated by the creation of quantum-based random number generators. Reliable random number generators are essential for encryption in cryptography. Pseudorandom number generators produce sequences of numbers that are not entirely unpredictable. However, these sequences lack recognizable patterns and, from the perspective of someone unaware of their nature, can be challenging to distinguish from those generated by truly random methods [128]. A reliable random number generator must meet two essential conditions. First, the user should understand how the numbers are generated to ensure the correct procedures are followed. Second, from an adversary's perspective, the system should function as a black box to prevent exploitation of any knowledge about its internal mechanisms. There can be deviations from the intended design of a random number generator due to imperfections, aging components, accidental failures, or interference from adversaries, leading to undetected biases [129,130].

Quantum-based systems enable the development of a secure random number generator in which the user remains unaware of the internal generation mechanism. In contrast, the adversary possesses a complete understanding of it [129]. In a recent study, two photons were prepared in an entangled state and sent to different remote measurement stations, where their polarizations were recorded. The measurement outcomes showed a strong correlation due to the photons' entanglement. This correlation can be detected experimentally using statistical criteria known as violations of Bell inequalities. The strongly correlated behavior of the two remote photons suggests that they could potentially be used to develop a faster-than-light communication device. However, since faster-than-light communication is impossible, this implies that the violations of Bell inequalities signal random measurement outcomes. These violations serve as an experimental marker of randomness. Because violations of Bell inequalities can only be verified by analyzing the statistics of the observed outputs, the verification process acts as a black-box test of randomness. Over many trials, the sequence of measurement outcomes should accumulate enough uncertainty to extract truly random bits through clever post-processing techniques [67,128].

Entropy and disorder help charges separate more quickly by increasing the likelihood of finding lower-energy sites farther apart. Entropy can lower the height of the potential barrier by increasing the distance between an electron and a hole. Charges cannot separate before they recombine. The role of entropy in charge separation becomes essential in the presence of disorder. The combination of entropy and energetic disorder reduces the barrier to the dissociation of charge-transfer states, making it easier to overcome and, in some cases, even eliminating it. This phenomenon occurs even with localized and thermalized charges [131-134].

These examples demonstrate that implementing quantum-based rules can generate randomness, thereby enhancing the efficiency of various processes. These ideas align with the CDP, highlighting the importance of randomness in system operations and the need to account for it.

### CDP-based use of quantum effects in biological systems

Using second-generation AI systems can enhance functions or correct malfunctions by reducing noise. These methods enable the quantification of variability signatures and their incorporation into treatment regimens. Researchers are currently studying various variability in biological systems, including heart rate variability, cytokine secretion variability, and genome-derived variability [25-42, 95].

Quantifying quantum randomness can provide a unique way to scale up random-based algorithms developed to correct the malfunctioning of biological systems [17]. These involve, for example, quantifying the signatures of random proton jumps in DNA or enzymatic reactions. Quantifying quantum-based randomness can provide data for the input layers of a second-generation AI algorithm [16]. As these CDP-based algorithms involve quantifying random observables from biological systems, the use of quantum-based parameters is proposed to enhance the accuracy of the input data and thereby improve the algorithms' performance, ultimately benefiting the clinical outcomes of subjects treated with these algorithms. It also provides a means for improving the accuracy of patient diagnosis and monitoring [52].

## Conclusion

The CDP may consider the quantum effects observed in biological systems. According to this principle, randomness and noise do not merely disrupt quantum effects; instead, they are essential for enhancing the quantum-dependent functions of these systems. The interactions among various external and internal noises, including those based on quantum principles, are inherent in all systems. These interactions determine the final structure and functions of the systems and are crucial for their proper operation. Future studies will investigate the potential application of quantum-based randomness to improve the efficiency of biological systems and establish the necessary mathematical framework for quantifying these phenomena.
